# Anterior hippocampal dysconnectivity in posttraumatic stress disorder: a dimensional and multimodal approach

**DOI:** 10.1038/tp.2017.12

**Published:** 2017-02-28

**Authors:** C G Abdallah, K M Wrocklage, C L Averill, T Akiki, B Schweinsburg, A Roy, B Martini, S M Southwick, J H Krystal, J C Scott

**Affiliations:** 1 Clinical Neurosciences Division, VA National Center for PTSD, US Department of Veterans Affairs, West Haven, CT, USA; 2 Department of Psychiatry, Yale University School of Medicine, New Haven, CT, USA; 3 Department of Psychiatry, Perelman School of Medicine, University of Pennsylvania, Philadelphia, PA, USA; 4 VISN4 Mental Illness Research, Education, and Clinical Center, Philadelphia VA Medical Center, Philadelphia, PA, USA

## Abstract

The anterior hippocampus (aHPC) has a central role in the regulation of anxiety-related behavior, stress response, emotional memory and fear. However, little is known about the presence and extent of aHPC abnormalities in posttraumatic stress disorder (PTSD). In this study, we used a multimodal approach, along with graph-based measures of global brain connectivity (GBC) termed functional GBC with global signal regression (*f*-GBCr) and diffusion GBC (*d*-GBC), in combat-exposed US Veterans with and without PTSD. Seed-based aHPC anatomical connectivity analyses were also performed. A whole-brain voxel-wise data-driven investigation revealed a significant association between elevated PTSD symptoms and reduced medial temporal *f*-GBCr, particularly in the aHPC. Similarly, aHPC *d*-GBC negatively correlated with PTSD severity. Both functional and anatomical aHPC dysconnectivity measures remained significant after controlling for hippocampal volume, age, gender, intelligence, education, combat severity, depression, anxiety, medication status, traumatic brain injury and alcohol/substance comorbidities. Depression-like PTSD dimensions were associated with reduced connectivity in the ventromedial and dorsolateral prefrontal cortex. In contrast, hyperarousal symptoms were positively correlated with ventromedial and dorsolateral prefrontal connectivity. We believe the findings provide first evidence of functional and anatomical dysconnectivity in the aHPC of veterans with high PTSD symptomatology. The data support the putative utility of aHPC connectivity as a measure of overall PTSD severity. Moreover, prefrontal global connectivity may be of clinical value as a brain biomarker to potentially distinguish between PTSD subgroups.

## Introduction

Posttraumatic stress disorder (PTSD) is a disabling mental illness, with limited treatment options and a pathophysiology that is not well understood.^1^ Identification of neural biomarkers underlying the symptomatology of PTSD may facilitate the development of novel efficacious therapeutics and could provide insight into the mechanisms underlying the disorder. Resting-state functional connectivity magnetic resonance imaging (*rs-fc*MRI), a technique for studying the covariance over time of regional brain activity, is a simple, yet powerful, method to investigate large-scale intrinsic brain networks.^2^ To date, *rs-fc*MRI studies in PTSD have primarily used seed-based hypothesis-driven approaches.^3, 4, 5, 6, 7, 8^ Recently developed graph theory-based approaches are being increasingly employed to identify, in a data-driven manner, neural network correlates of psychopathology.^2^ In this study, we used global brain connectivity (GBC)—also known as functional connectivity strength—to identify PTSD-related alterations in the resting covariance structure of brain activity in combat-exposed veterans with high PTSD symptoms. We first conducted a whole-brain data-driven analysis using functional GBC with global signal regression (*f*-GBCr), followed by dimensional and region of interest (ROI) analyses using *f*-GBCr, diffusion GBC (*d*-GBC) and tractography seed-based structural connectivity to confirm the data-driven findings and to examine the pattern and extent of dysconnectivity in PTSD.

Neuroimaging studies over the past 2 decades have identified a number of circuitry perturbations in PTSD patients.^1^ These alterations were primarily found in brain regions within the prefrontal cortex (PFC; for example, anterior cingulate and ventromedial PFC) and medial temporal lobe (for example, hippocampus (HPC) and amygdala). Together with preclinical data, such studies have encouraged the development of putative circuit-based models of PTSD, which propose altered activity and connectivity in critical brain regions as the underlying mechanism of PTSD abnormalities in stress response, fear conditioning and emotion regulation.^9, 10, 11, 12^ A complementary synaptic model of traumatic stress suggests that severe traumatic events, and the ensuing chronic stress experience, reduce synaptic connectivity in the PFC and HPC by reducing synaptic strength, spine density and dendritic arborization and length.^13, 14^ Supporting the preclinical data, a number of individual studies have reported PFC and HPC gray matter (GM) structural alterations in PTSD, which have been supported by systematic reviews and meta-analyses.^15, 16^ While prefrontal deficits appear to be mostly acquired post trauma,^17^ HPC structural alterations are believed to be both predisposing and acquired features.^17, 18^ Moreover, HPC structural deficits show a pattern of normalization following PTSD treatment.^19^

Recently, *f*-GBCr has been successfully used as a robust well-validated data-driven biomarker to identify brain regions with altered connectivity in neuropsychiatric disorders. Convergent evidence shows reduced PFC, but not medial temporal, *f*-GBCr across several disorders with strong contributions from chronic stress, including depression, bipolar disorder, schizophrenia and obsessive-compulsive disorder.^20, 21, 22, 23, 24^ *f*-GBCr is believed to reflect some of the variance in overall synaptic strength and connectivity in a brain region,^25^ and has been shown to positively correlate with brain energetics^26^ and normal brain functions.^27^ Pharmacological challenges have consistently shown PFC *f*-GBCr increases during states of presumable drug-induced glutamate neurotransmission surge.^23, 28, 29, 30^ Moreover, a pharmacological treatment known to reverse prefrontal stress-induced synaptic dysconnectivity in animals^31^ was found to reverse PFC *f*-GBCr reductions in depressed patients.^25^ However, it remains to be determined (a) whether *f*-GBCr alterations exist in PTSD; (b) to what extent these alterations are associated with anatomical dysconnectivity; and (c) whether such alterations are disorder-specific or stress-related abnormalities.

In this study, we conducted a whole-brain data-driven investigation examining the relationship between PTSD symptomatology, as measured by the Clinician Administered PTSD Scale (CAPS), and functional connectivity strength, as measured by *f*-GBCr. The data-driven analysis identified the medial temporal lobe, primarily the anterior hippocampus (aHPC), as a critical node for PTSD-related dysconnectivity. Surprisingly, there were no significant correlations between overall PTSD symptom severity and any brain region in the PFC. To substantiate and interrogate the data-driven findings, the whole-brain functional connectivity analyses were followed by targeted region-specific dimensional and multimodal investigations. First, we confirmed that aHPC *f*-GBCr dysconnectivity remained significant after controlling for hippocampal volume, depression severity, medication status and various clinical and demographic putative confounds. Second, to address concerns regarding global signal regression (GSR) and cluster-wise correction, two major controversies in *f*MRI field,^32, 33^ we determined the presence of aHPC dysconnectivity using an independent measure of anatomical dysconnectivity (*d*-GBC), an analysis based on ROI, and does not involve GSR. Third, considering the scarcity of studies employing dimensional models of PTSD and related psychopathology, we conducted an exploratory analysis examining the relationship between PFC *f*-GBCr and the four dimensions of PTSD symptomatology (that is, arousal, re-experiencing, numbing and avoidance^34^). Fourth, using probabilistic tractography and the aHPC as a seed, we identified the brain regions driving the aHPC dysconnectivity in veterans with high PTSD symptoms. Then, we explored the relationship between aHPC–PFC structural connectivity and each of the four PTSD symptom dimensions. It is important to note that the majority of previous investigations were conducted in case–control studies of individuals with a diagnosis of PTSD versus those without a diagnosis. This approach has the strength of instituting a large contrast; however, it could also create a potentially artificial dichotomization. In the current report we used a data-driven, hypothesis-free methodology along with a single-group dimensional approach. While the study findings may diverge from previous findings, the current report aims to capture the association between a continuum of biological abnormalities and clinical severity.

## Materials and methods

### Participants and clinical assessments

Seventy-one US Veterans between the age of 21 and 60 participated in this study, following an informed consent process and institutional approval of all study procedures. Study criteria required combat exposure and excluded the following comorbidities: psychotic disorder, bipolar disorder, learning disorder, attention deficit hyperactivity disorder, moderate or severe traumatic brain injury (TBI; using American Congress of Rehabilitation Medicine criteria^35^), epilepsy, brain tumor or other neurological disorders. Participants were also excluded if they were taking benzodiazepines or if they had an MRI contraindication. Given their high co-occurrence with PTSD in veterans, stable antidepressants and comorbid mild TBI, depression, anxiety and alcohol/substance disorders were not excluded to ensure the external validate of the study and the generalizability of the findings to the target population. These putative confounds were examined as covariates in *post hoc* analyses. The CAPS-IV was used to determine PTSD diagnosis and severity of symptoms.^36^ The Structured Clinical Interview for DSM-IV was completed to assess psychiatric comorbidities.^37^ Depression and anxiety symptoms were assessed using Beck Depression and Anxiety Inventories.^38, 39^ Combat exposure severity was assessed using the Combat Exposure Scale.^40^ Premorbid intellectual functioning was assessed using Wechsler Test of Adult Reading.^41^

### Neuroimaging methods

The imaging protocol included the following: (a) three high-resolution structural MRI (*s*MRI), (b) two 5 min *rs-fc*MRIs and (c) a diffusion MRI (*d*MRI) scan with a *b*-value of 1000 s mm^−^^2^ and 128 noncollinear encoding directions. A Siemens TIM Trio 3.0 Tesla magnet with a 32-channel head coil was used. MRI acquisition included: 2 x T1-weighted MPRAGE (voxel size = 1x1x1 mm; TR = 2530 ms; TE = 2.71 ms; Flip = 7°); 1 x T2-weighted (voxel size = 1x1x1 mm; TR = 3200 ms; TE = 419 ms; Flip = 120°); 2 x T2*-weighted BOLD resting state runs (voxel size = 3.4x3.4x3.4 mm; TR = 2000 ms; TE = 25 ms; Flip = 80° 145 frames); 1 diffusion weighted image (voxel size = 1.7x1.7x3 mm; TR = 7400 ms; TE = 115 ms; Flip = 90° b value = 1000 s/mm2; 128 directions). Details of *f*-GBCr methods were previously reported^25^ and are further described in the Supplemnetary Information. Briefly, following standard *rs-fc*MRI-preprocessing procedures, each voxel *f*-GBCr value was calculated as the average of its correlation with all other voxels in the brain GM. Processing of *d*MRI scans was performed using the *trac-all –prep –bedp* pipelines in Freesurfer,^42^ followed by seed-based probabilistic tractography using the FMRIB's Software Library (FSL) FMRIB's Diffusion Toolbox (FDT) (*probtrackx2*).^43^ *d*-GBC was calculated as the average of probabilistic anatomical connectivity between each voxel within the aHPC and all other voxels in the GM mask (see Supplementary Information).

### Statistical analyses

For details, please see Supplementary Information. Briefly, linear regressions were used to examine the relationship between CAPS total scores or dimension-specific score, and the following study biomarkers: *f*-GBCr, *d*-GBC and aHPC anatomical connectivity. Type I error correction was based on peak and cluster extent. All tests are two-tailed with significance set at *P*⩽0.05.

## Results

Demographic and psychiatric variables are presented in Supplementary Table S1. On average, participants had moderate level of PTSD symptoms. To alleviate concerns that the study findings are affected by head motion during scans, we examined the correlation between CAPS scores and motion parameters (see Supplemnetary Information) of *rs-fc*MRI (relative motion: *r*=0.01; *P*=0.95; absolute motion: *r*=0.05; *P*=0.70; % scrubbing: *r*=0.03; *P*=0.83) and *d*MRI (translation motion: *r*=0.12; *P*=0.33; rotation motion: *r*=–0.01; *P*=0.93). Similarly, the study secondary measures (numbing, avoidance, arousal and re-experiencing) did not correlate with motion parameters (all *P*-values>0.1).

### Whole-brain functional connectivity

**Figure 1:**
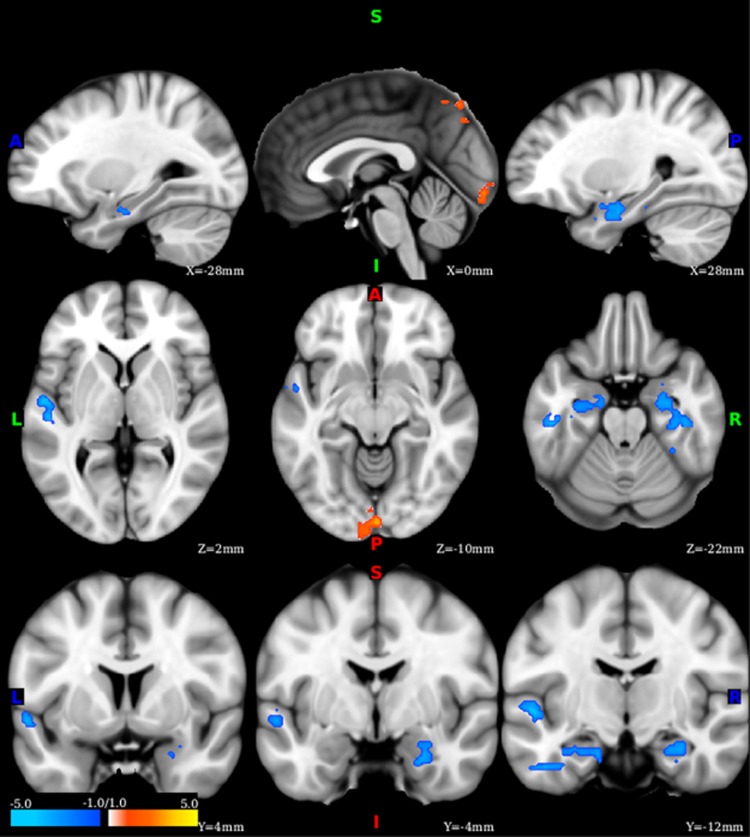
Functional dysconnectivity in posttraumatic stress disorder (PTSD). Voxel-wise whole-brain correlations between PTSD severity, as measured by the Clinician Administered PTSD Scale (CAPS) and functional global brain connectivity with global signal regression. The color bar depicts the *z-*values of the negative (blue) and positive (yellow–red) correlations.

Following correction for multiple comparisons, the whole-brain data-driven analysis revealed large clusters of significant negative correlations between CAPS scores and *f*-GBCr in medial temporal regions, particularly overlapping with the aHPC (Figure 1 and Table 1). Additional significant clusters were located in the left superior temporal gyrus (negative correlations), and midline occipital and precuneus areas (positive correlations). Considering the critical role of the aHPC in the regulation of affect and memory, we used the aHPC as a ROI in a set of secondary analyses to determine the extent of the discovered dysconnectivity. In each individual, we extracted the voxels overlapping between the anatomical hippocampus and the clusters of significant correlation between CAPS and *f*-GBCr; this provided a subject-specific aHPC ROI.

Although our *f*-GBCr methods minimize the effects of GM variability between participants (for example, using a study-specific GM mask based on the 95% overlap across subjects; see Supplemnetary Information), we did examine the effect of hippocampal volume on aHPC *f*-GBCr to rule out the possibility that the observed dysconnectivity is primarily driven by the well-documented hippocampal reduction in PTSD. We found no significant correlation between hippocampal volume and *f*-GBCr (*r*=0.18; *P*=0.15). Covarying for hippocampal volume, a partial correlation analysis showed significant negative correlation between aHPC *f*-GBCr and CAPS (*r*=–0.41; *P*=0.001). Similarly, the correlation between aHPC *f*-GBCr and CAPS remained significant (*P*<0.05) after controlling for each of the following variables' age, gender, intelligence, education, combat severity, depression, anxiety, medication status, TBI and alcohol/substance comorbidities (Supplementary Table S2).

### Dimension-specific PFC functional connectivity

**Figure 2:**
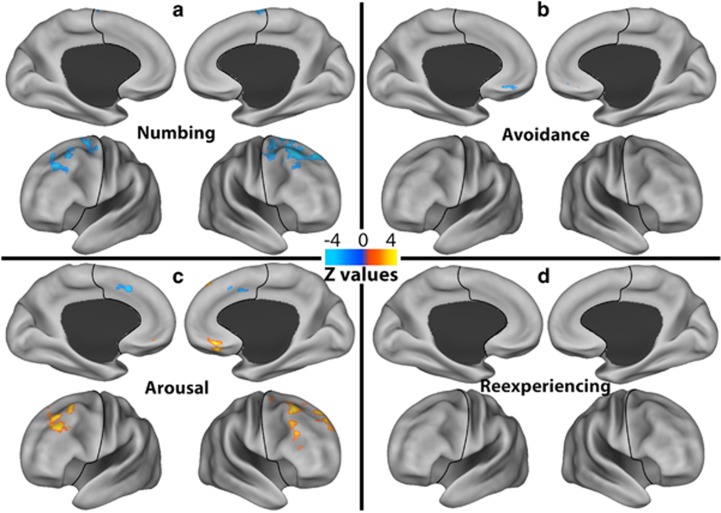
Dimension-specific prefrontal dysconnectivity. Voxel-wise correlations between functional global brain connectivity with global signal regression within the prefrontal cortex and the severity of the four posttraumatic stress disorder dimensions ((**a**) numbing; (**b**) avoidance; (**c**) arousal; (**d**) re-experiencing). The prefrontal cortex region is labeled with a black line. The color bar depicts the *z*-values of the negative (blue) and positive (yellow–red) correlations.

Comparable to previous findings in depressive disorders,^20, 21, 25^ the depression-like dimensions showed significant negative correlations with *f*-GBCr in the lateral PFC (numbing), and left ventromedial and dorsal PFC (avoidance; Figure 2 and Table 1). In contrast, arousal severity showed primarily positive correlations with *f*-GBCr in the lateral PFC and right ventromedial PFC, with one cluster of negative correlation with *f*-GBCr in the dorsomedial PFC. Re-experiencing severity did not correlate with *f*-GBCr.

### Anatomical dysconnectivity

**Figure 3:**
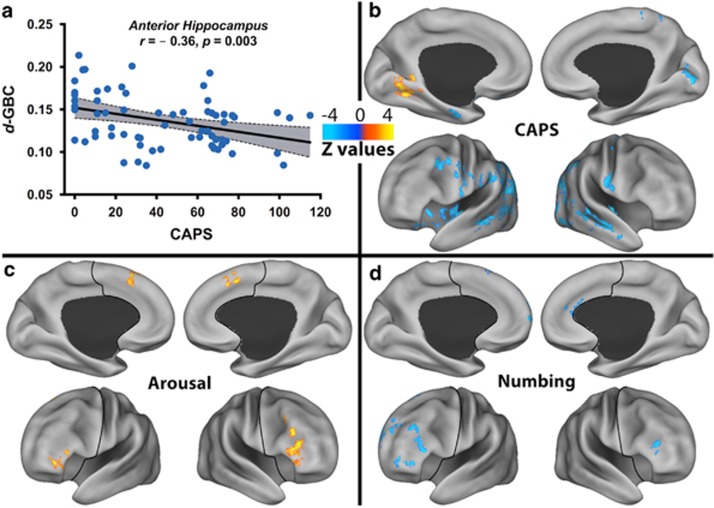
Anatomical dysconnectivity in posttraumatic stress disorder (PTSD). (**a**) Scatter plot depicting the correlation between PTSD severity, as measured by the Clinician Administered PTSD Scale (CAPS) and anterior hippocampal (aHPC) diffusion global brain connectivity (*d*-GBC). The gray area is the 95% confidence band of the best-fit line. (**b**) Voxel-wise whole-brain correlations between PTSD severity and aHPC tractography seed-based connectivity. (**c**, **d**) Voxel-wise correlations between aHPC tractography seed-based connectivity within the prefrontal cortex and the severity of the PTSD dimensions ((**c**) arousal; (**d**) numbing; avoidance and re-experiencing had no significant correlations). The prefrontal cortex region is labeled with a black line. The color bar depicts the *z-*values of the negative (blue) and positive (yellow–red) correlations.

To determine the presence of aHPC anatomical dysconnectivity, we first examined the relationship between CAPS and *d*-GBC, followed by seed-based anatomical connectivity using the aHPC as seed and the whole-brain GM as target. Similar to *f*-GBCr, we found a significant negative correlation between aHPC *d*-GBC and CAPS (*r*=–0.36; *P*=0.003; Figure 3a). *d*-GBC did not correlate with hippocampal volume (*P*=0.37). Moreover, the correlation between aHPC *d*-GBC and CAPS remained significant (*P*<0.05) after controlling for each of the following variables: hippocampal volume, age, gender, intelligence, education, combat severity, depression, anxiety, medication status, TBI and alcohol/substance comorbidities (Supplementary Table S2).

Following correction for multiple comparisons, the whole-brain seed-based analysis revealed widespread negative correlations between CAPS and the aHPC connectivity with the insula, and with clusters within the temporal, parietal and occipital lobes, noticeably sparing the PFC area. One positive correlation cluster was found in the lingual area (Figure 3b). A *post hoc* PFC voxel-wise anatomical connectivity analysis demonstrated significant positive correlations between arousal severity and the aHPC connectivity with the lateral and dorsomedial PFC (Figure 3c), but negative correlations between numbing severity and the aHPC connectivity with the lateral PFC and right rostral anterior cingulate (Figure 3d). There were no significant correlations with avoidance and re-experiencing dimensions.

## Discussion

The results identified PTSD-specific functional dysconnectivity in the medial temporal cortex, primarily the aHPC. Anatomical dysconnectivity was also demonstrated in the aHPC. Both functional and anatomical aHPC dysconnectivity were independent of the effects of medication and TBI status, depression and anxiety severity or comorbid substance use disorders. Similarly, aHPC dysconnectivity measures were not affected by HPC volume. Surprisingly, in contrast to findings in several stress-related disorders, PTSD severity showed no correlations with *f*-GBCr in any brain region within the PFC. However, follow-up exploratory analyses revealed that the severity of depression-like dimensions (for example, numbing) negatively correlated with PFC *f*-GBCr and aHPC–PFC anatomical connectivity. In contrast, hyperarousal symptoms were positively associated with PFC *f*-GBCr and aHPC–PFC anatomical connectivity. Together, the findings support the potential utility of aHPC connectivity as a biomarker of overall PTSD severity, which might be valuable in drug development as a biomarker of target validation. In addition, PFC connectivity may prove of clinical utility as a biological brain measure to distinguish between PTSD subgroups, particularly between patients with prominent arousal or numbing symptoms. Finally, comparable to previous findings of increased parietal and occipital *f*-GBCr in depressed patients,^20, 25^ we found a positive correlation between PTSD severity and clusters within the parietal/occipital area. This anterior–posterior dichotomy is believed to be the result of reduced rostral input, leading to increased caudal activity.^44, 45^ For example, treatment-resistant depression patients showed significant functional dysconnectivity between PFC and caudal brain structures at baseline. However, ketamine treatment, which increased PFC *f*-GBCr, led to increased PFC–occipital/parietal connectivity and simultaneous reduction in occipital/parietal *f*-GBCr.^25^

The HPC has a major role in several brain functions critical to PTSD psychopathology. While the majority of the PTSD literature has focused on the full HPC, accumulating evidence suggests a wide structural and functional variation along the longitudinal axis. Early studies suggested a binary division between the anterior 33% and the posterior 66% of the HPC, with the latter mostly involved in spatial memory.^46^ However, findings over the past two decades have presented a more complex picture supporting a model in which the HPC is divided into anterior (25%), intermediate (50%) and posterior (25%) subregions.^47^ The aHPC appears to have a more central role in anxiety-related behavior, stress response, emotional memory and fear. The posterior hippocampus (pHPC) has a more primary role in spatial memory and navigation, pattern separation and contextual fear conditioning.^48^ A number of HPC structures (molecular/anatomical) and functions follow a discrete transition between subregions. For example, unconditioned fear and amygdala connectivity are limited to the aHPC and intermediate hippocampus (iHPC). However, other functions show a gradient, non-discrete, transition between HPC subregions, with a pattern of high functional sensitivity but low specificity in the aHPC, that is believed to be evolutionarily advantageous to detect danger, but more detailed and contextually rich processing in the pHPC.^48^ For example, the aHPC place cells are sparse with low spatial selectivity compared to the pHPC, which has considerably higher density and much better spatial resolution.^48^ Similarly, detailed autobiographical and spatial memories activate the pHPC, while the aHPC is associated with ‘gist-like’ memory.^49^ Considering their proposed functions, both the aHPC and pHPC are hypothesized to contribute to PTSD pathophysiology, with pHPC alterations particularly involved in contextual fear conditioning.^6, 50, 51^

The aHPC findings in the current study have several implications. First, they support the sensitivity of *f*-GBCr as a robust data-driven biomarker to identify psychopathology-related dysconnectivity in PTSD. In addition, considering the lack of comparable medial temporal *f*-GBCr abnormalities in previously examined neuropsychiatric disorders,^20, 21, 22, 23, 24^ the data also suggest potential specificity of aHPC alterations to PTSD symptomatology, although such specificity remains to be demonstrated in future studies directly comparing aHPC *f*-GBCr across disorders. Second, although our study design could not differentiate between predisposing and acquired abnormalities, we speculate that both of these non-mutually exclusive possibilities likely contributed to the discovered aHPC dysconnectivity. At least one study has suggested that HPC volumetric abnormality predates trauma exposure and PTSD psychopathology, while several converging lines of evidence demonstrate trauma- and stress-induced HPC abnormalities, confirming the acquired nature of at least part of the PTSD-related HPC pathology that may normalize following treatment.^17, 19^ In this model, predisposing aHPC deficits further exacerbated by traumatic stress leads to circuitry perturbations, precipitating the constellation of PTSD symptomatology, which in turn further exacerbates the stress-related HPC pathology.

Another implication of the aHPC findings is the localization of dysconnectivity to the aHPC instead of the whole HPC, as well as the correlational specificity of the aHPC–PFC dysconnectivity to hyperarousal (positive association) versus numbing symptomatology (negative association). Similarly, the PFC *f*-GBCr alterations were dimension-specific, showing opposing relationship with different dimensions. This regional and dimensional specificity of abnormalities may provide putative explanations for some of the inconsistencies in the literature, which have primarily studied PTSD as binary disorder of subjects with or without PTSD and/or investigated the full HPC as one anatomical and functional region. PTSD studies examining the aHPC subregion are relatively scarce. One study showed significant aHPC volume reduction in PTSD, but a later study failed to replicate the aHPC findings.^50, 52^ A recent functional connectivity study reported a trend of reduced connectivity between the aHPC and dorsomedial PFC in patients with PTSD or generalized anxiety disorder, and no connectivity differences between PTSD and generalized anxiety disorder groups. However, in an effort to avoid potential overlap with the amygdala, the aHPC seed may have been mostly located in the anterior end of the iHPC instead of the anterior 25% portion of the HPC.^6^

### Limitations and strengths

Similar to other cross-sectional approaches, it is important to underscore that correlations do not necessarily imply causation. Thus, the study cannot differentiate risk factors for PTSD from consequences of trauma. In addition, the data do not determine whether structural alterations preceded functional dysconnectivity, or vice versa. In addition, although known putative confounds were assessed in partial correlation analyses, it is plausible that other unknown confounds might have contributed to study findings. Another potential limitation is that common comorbidities and stable treatment with antidepressants were not excluded; however, they were tested in *post hoc* analyses. These were permitted to ensure the external validity and generalizability of the findings to the target veteran population. Finally, the veterans were primarily male participants.

Among the strengths of the study is the data-driven, hypothesis-free, explore-then-validate methodolgoy along with a single-group dimensional approach. Another strength is the use of high-quality *state-of-the-art* well-validated multimodal neuroimaging methods, along with rigorous control procedures. The study protocol included two T1s and one T2 high-resolution scans to ensure quality structural segmentation. Two *rs-fc*MRI scans were acquired separately to minimize fatigue and 128 encoding directions were used to enhance the *d*MRI tractography estimates. We employed well-validated *f*-GBCr methods that we previously showed to be sensitive to psychopathology and treatment.^20, 25^ Finally, the use of *d*MRI ROI-based tractography was a vital addition, not only to confirm the putative presence of anatomical dysconnectivity, but also to alleviate concerns related to common *rs-fc*MRI controversies, including GSR and correction for type I error.^32, 33^

## Figures and Tables

**Table 1:** *f*-GBCr correlations with PTSD symptoms

*Region*	*Side*	*Coordinates (peak)*	*Cluster size (mm*^*3*^)	*Correlation*
*CAPS*				
Medial temporal	L	−16, −12, −24	634	Negative
Medial temporal	R	42, −24, −22	614	Negative
Superior temporal	L	−52, −8, 0	438	Negative
Occipital	L R	2, −90, −10	308	Positive
Precuneus	L R	−4, −74, 52	292	Positive
				
*Numbing*				
Lateral PFC	R	30, 18, 46	3052	Negative
Lateral PFC	L	−36, −6, 62	1196	Negative
Lateral PFC	L	−28, 28, 38	406	Negative
				
*Avoidance*				
Dorsal PFC	L	−28, 38, 46	254	Negative
Ventromedial PFC	L	0, 36, −20	204	Negative
				
*Arousal*				
Lateral PFC	R	34, 24, 54	1348	Positive
Lateral PFC	L	−32, 34, 44	1108	Positive
Ventromedial PFC	R	10, 32, −22	404	Positive
Lateral PFC	R	46, 26, 36	314	Positive
Lateral PFC	R	20, 50, 40	314	Positive
Dorsal PFC	R	8, 46, 48	308	Positive
Dorsomedial PFC	L R	0, 12, 38	280	Negative
				
*Re-experiencing*				
None				

Abbreviations: CAPS, Clinician Administered PTSD Scale; *f*-GBCr, functional global brain connectivity with global signal regression; L, left; PFC, prefrontal cortex; PTSD, posttraumatic stress disorder; R, right.
