# Supplementary material for: Anterior hippocampal dysconnectivity in posttraumatic stress disorder: a dimensional and multimodal approach
Source: Transl Psychiatry. 2017 Feb 28;7(2):e1045–. doi: 10.1038/tp.2017.12 (PMC5545643; doi:10.1038/tp.2017.12)
Supplement: Supplementary Information [file tp201712x1.docx]

**Supplements**

***Neuroimaging Acquisition***

A Siemens TIM Trio 3.0 Tesla magnet with a 32-channel head coil was used. MRI acquisition included: 2 x T1-weighted MPRAGE (voxel size = 1x1x1 mm; TR = 2530 ms; TE = 2.71 ms; Flip = 7°); 1 x T2-weighted (voxel size = 1x1x1 mm; TR = 3200 ms; TE = 419 ms; Flip = 120°); 2 x T2*-weighted BOLD resting state runs (voxel size = 3.4x3.4x3.4 mm; TR = 2000 ms; TE = 25 ms; Flip = 80°; 145 frames); 1 diffusion weighted image (voxel size = 1.7x1.7x3 mm; TR = 7400 ms; TE = 115 ms; Flip = 90°; b value = 1000 s/mm^2^; 128 directions).

***Neuroimaging Processing and Analyses***

All image processing and quality control procedures were conducted while blinded to the participants’ demographic and clinical characteristics.

*Structural Segmentation & Parcellation*: The publically available software package Freesurfer ([http://surfer.nmr.mgh.harvard.edu](http://surfer.nmr.mgh.harvard.edu/); v5.3) was used for *s*MRI image processing, cortical surface reconstruction, and volumetric segmentation, as previously described ^1^. Briefly, 2 x T1 and 1 T2 were included to improve motion correction and delineation of the pial surface, respectively. The *recon-all* pipeline included imaging intensity normalization, removal of non-brain tissue, segmentation of the gray/white matter (GM/WM) and subcortical volumetric structures (including hippocampus), tessellation of the GM WM boundary, automated topology correction, spherical surface-based intersubject registration based on curvature (sulcus and gyri), and automated parcellation of cortical regions. Further technical details of these procedures are as described previously {Fischl, 2000 #36;Dale, 1999 #42;Fischl, 1999 #41;Fischl, 1999 #39;Jovicich, 2006 #11;Han, 2006 #6;Fischl, 2004 #27;Segonne, 2004 #24;Fischl, 2004 #22;Fischl, 2002 #32;Fischl, 2001 #33;Dale, 1993 #43;Dale, 1999 #1405;Fischl, 2002 #1169;Fischl, 1999 #1406;Reuter, 2010 #1407}^2-5^. Freesurfer post-processing quality checking routines were followed; no manual corrections were necessary. Individual anatomical prefrontal cortex (PFC) masks (labeled in Fig. 2 & 4) were created based on the Freesurfer segmentation of the high resolution T1/T2 scans. Each PFC mask included the following right and left regions: caudal anterior cingulate, caudal middle frontal, lateral orbitofrontal, medial orbitofrontal, pars opercularis, pars orbitalis, pars triangularis, rostral anterior cingulate, rostral middle frontal, superior frontal, and frontal pole. The subject specific anterior hippocampal (aHPC) seed mask (region of interest; ROI) was based on the overlap between the anatomical hippocampus and the functional clusters showing significant correlation with CAPS.

*Functional Preprocessing & f-GBCr*: Each 5 min run was processed separately. The 2 runs per session were averaged prior to 2^nd^ level group analyses. Details of *f*-GBCr processing and analysis methods were previously described ^6-15^. Briefly, the preprocessing of each *rs-fc*MRI included brain extraction, motion correction, slice-time correction, spatial smoothing (FWHM 5 mm), high-pass temporal filtering (100 s), nonlinear registration of structural images to a standard Montreal Neurological Institute (MNI) template (2x2x2 mm), boundary-based registration (BBR) of rs-*fc*MRI to high-resolution T1 images, and regression of motion parameters, cerebrospinal fluid (CSF), white matter, and global brain signal, and their 1^st^ derivatives. In addition, motion scrubbing, as per Power et al. ^16^, was completed prior to *f*-GBCr calculation.

Time series were extracted from all voxels within each individual’s anatomically defined whole-brain GM mask. Matrices of pairwise Pearson correlation coefficients of all GM voxels were generated, and then transformed to Fisher z values. For each voxel, *f*-GBCr is calculated as the normalized average across those Fisher z values, which generates a map for each subject where each voxel value represents the functional connectivity strength of that voxel with the rest of the brain. In graph theory terms, *f*-GBCr (also known as Functional Connectivity Strength; FCS ^17^) is considered a measure of nodal strength of a voxel in the whole brain network – determining brain hubs and examining the coherence between a local region and the rest of the brain ^18^.

All processing and analyses were conducted in the subject functional space, except for 2^nd^ level group analyses (MNI space; 2x2x2 mm). The study specific GM mask for *rs-fc*MRI analyses was created based on voxels with 95% overlap across all participants. All included scans passed the following quality control criteria: no BOLD run with a single frame movement greater than 1 functional voxel and no motion scrubbing of more than 50% of each run. Of study participants, 68 had successful functional scans. To further delineate the distribution of *f*-GBCr alteration in the aHPC and to investigate the effects of potential confounds, we extracted the average aHPC *f*-GBCr for each participant, which was used as an ROI in post-hoc analyses to examine the effects of several demographic and clinical covariates.

*Diffusion Processing & d-GBC*: Preprocessing and modeling of diffusion parameters were performed using *trac-all* pipeline in Freesurfer ^19^. The processing included estimation of motion ^20^, eddy current and head motion corrections, brain extraction, BBR registration of low-b diffusion (b = 0) to the high resolution images, registration of structural images to MNI template (1x1x1 mm), least-squares tensor estimation (*dtifit*), and the ball-and-stick model fit assuming one isotropic and two anisotropic compartments (*bedpostx*) ^21^. The multi-fiber probabilistic distributions per voxel allowed the tracing of pathways in regions with fiber crossing ^22^. Probabilistic tractography was performed by propagating 5000 streamline samples from each voxel within the aHPC seed through the probabilistic distributions, using a whole brain mask. The number (x) of streamlines passing through each brain voxel was log transformed [y = log (1 + x)], which generates a map for each subject where each voxel value (y) represents an estimate of the probability of anatomical connection to the seed. Individual aHPC *d*-GBC was calculated as the normalized average anatomical connectivity between the aHPC and all GM voxels. All processing and analyses were conducted in the subject diffusion space, except for 2^nd^ level group analyses (MNI space; 1x1x1 mm). The study specific GM and PFC masks for *d*MRI analyses were based on the Freesurfer segmentation, including 2 mm dilation of the cortical WM surface. Of study participants, 67 had successful diffusion scans.

***Statistical Analyses***

The distribution of outcome measures was examined using probability plots and test statistics. Transformations and non-parametric tests were used as necessary. Estimates of variation are provided as standard error of the mean (SEM). Considering that this is a first-in-humans study to examine functional and anatomical GBC in PTSD, the sample size was estimated based on prior stress-related disorders showing significant central effects in cohorts of 45 to 82 subjects ^6, 7^.

Voxel-wise *rs-fc*MRI and *d*MRI analyses used linear regressions, followed by correction for Type I error as follow: we first estimated smoothness through AFNI’s 3dFWHMx and then used 3dClustSim with 10000 Monte Carlo simulations, uncorrected z value > 2.3, and corrected *α* = 0.05 ^23^. This procedure generated the following cluster level contiguous voxels thresholds: 120 for whole brain and 99 for PFC *rs-fc*MRI analyses (voxel size 2x2x2 mm); 562 for whole brain and 328 for PFC *d*MRI analyses (voxel size 1x1x1 mm). To determine the effects of putative confounds, we first extracted the average *f*-GBCr of the aHPC of each subject (see Neuroimaging section above). Then we conducted partial correlation analyses between CAPS and aHPC *f*-GBCr controlling for each of the following variables: age, gender, WTAR, education, BDI, BAI, CES, and TBI and alcohol/substance use disorder status. To determine the relationship between PFC connectivity and depression-like PTSD symptom dimensions, CAPS items were divided into four dimensions (i.e., numbing, avoidance, arousal, and reexperiencing), as previously described ^24^. Then, we conducted PFC voxel-wise regression analyses correlating connectivity measures (*f*-GBCr or aHPC-PFC anatomical connectivity) with each of the PTSD dimensions, while controlling for the remaining 3 dimensions.

To assess the robustness of the woxel-wise results, we have repeated these analyses using a *p* value of 0.01. As shown in Fig. S1-3, the results were comparable to those of the primary analysis shown in Fig. 1-3.

**
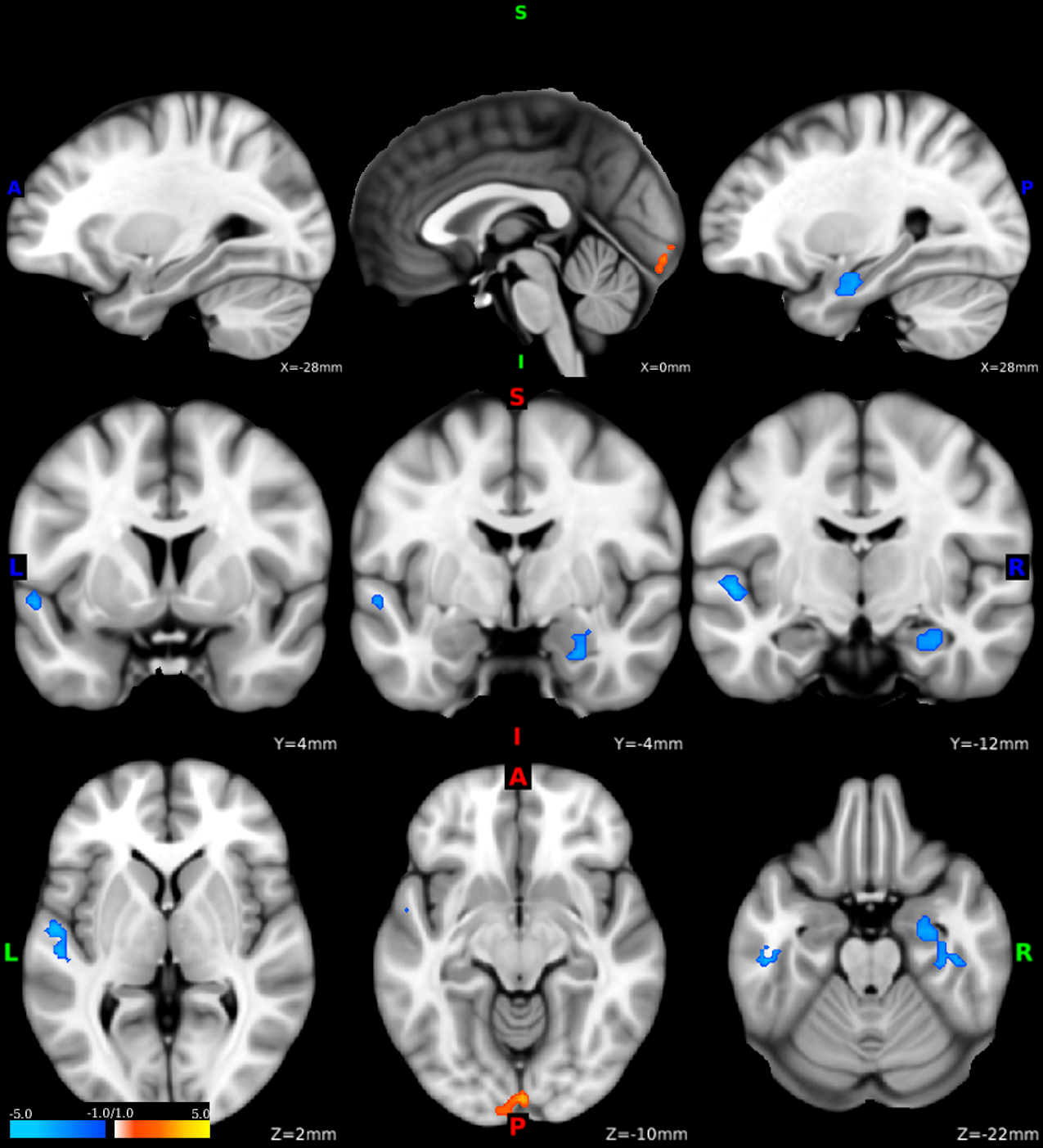
**

**Figure S1**. **Functional Dysconnectivity in PTSD**. Voxel-wise whole-brain correlations between PTSD severity, as measured by the Clinician Administered PTSD Scale (CAPS), and functional Global Brain Connectivity with global signal regression (*f*-GBCr). The color bar depicts the z values of the negative (blue) and positive (yellow-red) correlations.

**
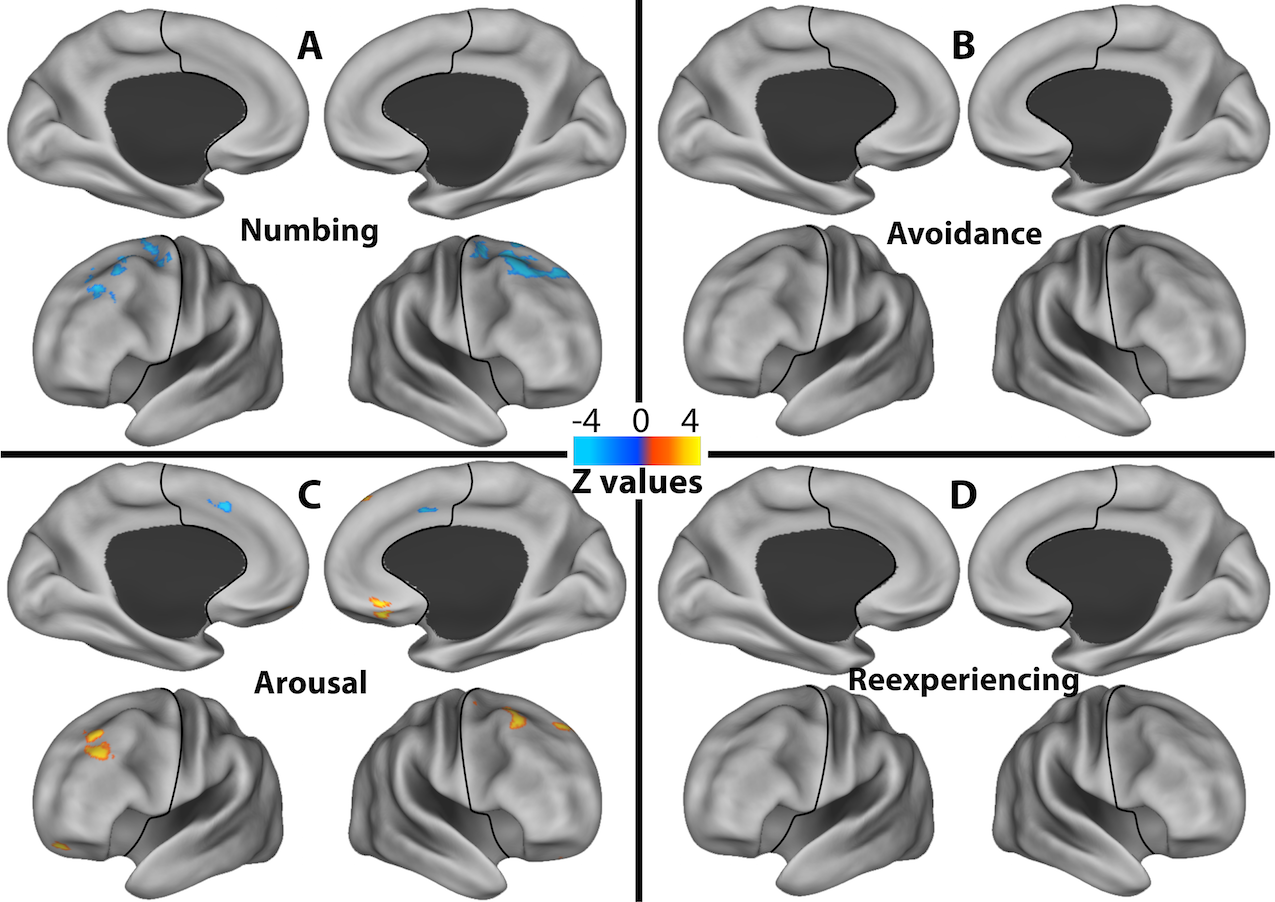
**

**Figure S2**. **Dimension specific prefrontal dysconnectivity**. Voxel-wise correlations between functional Global Brain Connectivity with global signal regression (*f*-GBCr) within the prefrontal cortex and the severity of the four PTSD dimensions (A – Numbing; B – Avoidance; C – Arousal; D – Reexperiencing). The prefrontal cortex region is labeled with a black line. The color bar depicts the z values of the negative (blue) and positive (yellow-red) correlations.

**
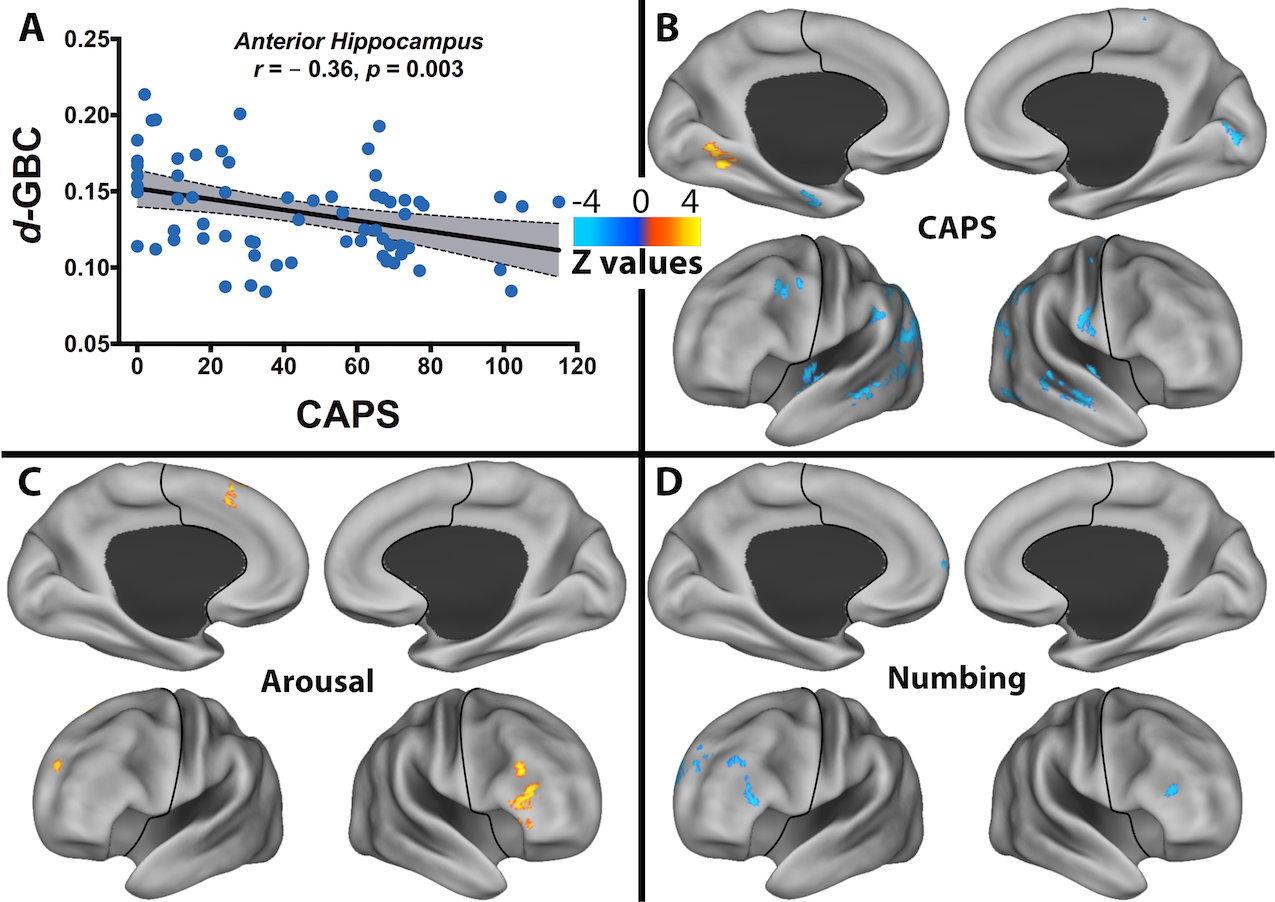
**

**Figure S3. Anatomical Dysconnectivity in PTSD**. A – Scatter plot depicting the correlation between PTSD severity, as measured by the Clinician Administered PTSD Scale (CAPS), and anterior hippocampal (aHPC) diffusion Global Brain Connectivity (*d*-GBC). The grey area is the 95% confidence band of the best-fit line. B – Voxel-wise whole-brain correlations between PTSD severity and aHPC tractography seed-based connectivity. C, D – Voxel-wise correlations between aHPC tractography seed-based connectivity within the prefrontal cortex and the severity of the PTSD dimensions (C – Arousal; D – Numbing; Avoidance and Reexperiencing had no significant correlations). The prefrontal cortex region is labeled with a black line. The color bar depicts the z values of the negative (blue) and positive (yellow-red) correlations.

**Table S1. Demographic and clinical characteristics.**

|  | **Mean ±*SEM* or %** |
| --- | --- |
| Age (years) | 34.4 ± 1.1 |
| Females | 11% |
| WTAR | 103 ± 1.0 |
| Education (years) | 14.0 ± 0.2 |
| CAPS | 44.7 ± 3.6 |
| CES | 18.0 ± 1.2 |
| BDI | 18.9 ± 1.5 |
| BAI | 13.4 ± 1.3 |
| DSM-IV Axis I | 65% |
| PTSD | 51% |
| MDD | 18% |
| SUD | 23% |
| Anxiety | 7% |
| Medicated | 33% |
| Mild TBI | 59% |

Abbreviations – SEM: Standard Error of Means; WTAR: Wechsler Test of Adult Reading; CAPS: Clinician Administered PTSD Scale for the DSM-IV; CES: Combat Exposure Scale; BDI: Beck Depression Inventory; BAI: Beck Anxiety Inventory; PTSD: Posttraumatic Stress Disorder; MDD: Major Depressive Disorder; SUD: Substance/Alcohol Use Disorder; Anxiety: Panic Disorder, Generalized Anxiety Disorder, Obsessive Compulsive Disorder; TBI: Traumatic Brain Injury

**Table S2. CAPS correlations with anterior hippocampal (aHPC) *f*-GBCr and *d*-GBCr.**

| Covariate | **aHPC *f*-GBCr *** | **aHPC *d*-GBCr *** | **Average **** |
| --- | --- | --- | --- |
| None | –0.43 | –0.36 | –0.40 |
| Age | –0.44 | –0.38 | –0.41 |
| Gender | –0.44 | –0.37 | –0.41 |
| WTAR | –0.43 | –0.37 | –0.40 |
| Education | –0.42 | –0.36 | –0.39 |
| CES | –0.38 | –0.41 | –0.40 |
| BDI | –0.32 | –0.37 | –0.35 |
| BAI | –0.30 | –0.48 | –0.39 |
| Medicated | –0.36 | –0.39 | –0.38 |
| TBI | –0.43 | –0.38 | –0.41 |
| SUD | –0.46 | –0.35 | –0.41 |

*Values are the coefficients of the bivariate correlations between CAPS and each of the aHPC connectivity measures. All correlations were statistically significant with *p* < 0.05. **Values are the average correlation coefficients between CAPS and aHPC connectivity. Abbreviations – CAPS: Clinician Administered PTSD Scale for the DSM-IV; *f*-GBCr: functional global brain connecitivy with global signal regression; *d*-GBC: diffusion global brain connectivity; WTAR: Wechsler Test of Adult Reading; CES: Combat Exposure Scale; BDI: Beck Depression Inventory; BAI: Beck Anxiety Inventory; TBI: Traumatic Brain Injury; SUD: Substance/Alcohol Use Disorder;

**References**:

1. Abdallah CG, Coplan JD, Jackowski A, Sato JR, Mao X, Shungu DC*, et al*. A pilot study of hippocampal volume and N-acetylaspartate (NAA) as response biomarkers in riluzole-treated patients with GAD. *Eur Neuropsychopharmacol* 2013; **23**(4)**:** 276-284.

2. Dale AM, Fischl B, Sereno MI. Cortical surface-based analysis. I. Segmentation and surface reconstruction. *Neuroimage* 1999; **9**(2)**:** 179-194.

3. Fischl B, Salat DH, Busa E, Albert M, Dieterich M, Haselgrove C*, et al*. Whole brain segmentation: automated labeling of neuroanatomical structures in the human brain. *Neuron* 2002; **33**(3)**:** 341-355.

4. Fischl B, Sereno MI, Dale AM. Cortical surface-based analysis. II: Inflation, flattening, and a surface-based coordinate system. *Neuroimage* 1999; **9**(2)**:** 195-207.

5. Reuter M, Rosas HD, Fischl B. Highly accurate inverse consistent registration: a robust approach. *Neuroimage* 2010; **53**(4)**:** 1181-1196.

6. Abdallah CG, Averill LA, Collins KA, Geha P, Schwartz J, Averill C*, et al*. Ketamine Treatment and Global Brain Connectivity in Major Depression. *Neuropsychopharmacology* 2016.

7. Murrough JW, Abdallah CG, Anticevic A, Collins KA, Geha P, Averill LA*, et al*. Reduced global functional connectivity of the medial prefrontal cortex in major depressive disorder. *Hum Brain Mapp* 2016.

8. Anticevic A, Brumbaugh MS, Winkler AM, Lombardo LE, Barrett J, Corlett PR*, et al*. Global prefrontal and fronto-amygdala dysconnectivity in bipolar I disorder with psychosis history. *Biol Psychiatry* 2013; **73**(6)**:** 565-573.

9. Anticevic A, Corlett PR, Cole MW, Savic A, Gancsos M, Tang Y*, et al*. N-methyl-D-aspartate receptor antagonist effects on prefrontal cortical connectivity better model early than chronic schizophrenia. *Biol Psychiatry* 2015; **77**(6)**:** 569-580.

10. Anticevic A, Hu S, Zhang S, Savic A, Billingslea E, Wasylink S*, et al*. Global resting-state functional magnetic resonance imaging analysis identifies frontal cortex, striatal, and cerebellar dysconnectivity in obsessive-compulsive disorder. *Biol Psychiatry* 2014; **75**(8)**:** 595-605.

11. Anticevic A, Hu X, Xiao Y, Hu J, Li F, Bi F*, et al*. Early-course unmedicated schizophrenia patients exhibit elevated prefrontal connectivity associated with longitudinal change. *J Neurosci* 2015; **35**(1)**:** 267-286.

12. Cole MW, Anticevic A, Repovs G, Barch D. Variable global dysconnectivity and individual differences in schizophrenia. *Biol Psychiatry* 2011; **70**(1)**:** 43-50.

13. Cole MW, Yarkoni T, Repovs G, Anticevic A, Braver TS. Global connectivity of prefrontal cortex predicts cognitive control and intelligence. *J Neurosci* 2012; **32**(26)**:** 8988-8999.

14. Driesen NR, McCarthy G, Bhagwagar Z, Bloch M, Calhoun V, D'Souza DC*, et al*. Relationship of resting brain hyperconnectivity and schizophrenia-like symptoms produced by the NMDA receptor antagonist ketamine in humans. *Mol Psychiatry* 2013; **18**(11)**:** 1199-1204.

15. Driesen NR, McCarthy G, Bhagwagar Z, Bloch MH, Calhoun VD, D'Souza DC*, et al*. The impact of NMDA receptor blockade on human working memory-related prefrontal function and connectivity. *Neuropsychopharmacology* 2013; **38**(13)**:** 2613-2622.

16. Power JD, Barnes KA, Snyder AZ, Schlaggar BL, Petersen SE. Spurious but systematic correlations in functional connectivity MRI networks arise from subject motion. *Neuroimage* 2012; **59**(3)**:** 2142-2154.

17. Liang X, Zou Q, He Y, Yang Y. Coupling of functional connectivity and regional cerebral blood flow reveals a physiological basis for network hubs of the human brain. *Proc Natl Acad Sci U S A* 2013; **110**(5)**:** 1929-1934.

18. Cole MW, Pathak S, Schneider W. Identifying the brain's most globally connected regions. *NeuroImage* 2010; **49**(4)**:** 3132-3148.

19. Yendiki A, Panneck P, Srinivasan P, Stevens A, Zollei L, Augustinack J*, et al*. Automated probabilistic reconstruction of white-matter pathways in health and disease using an atlas of the underlying anatomy. *Frontiers in neuroinformatics* 2011; **5:** 23.

20. Yendiki A, Koldewyn K, Kakunoori S, Kanwisher N, Fischl B. Spurious group differences due to head motion in a diffusion MRI study. *Neuroimage* 2013; **88C:** 79-90.

21. Behrens TE, Woolrich MW, Jenkinson M, Johansen-Berg H, Nunes RG, Clare S*, et al*. Characterization and propagation of uncertainty in diffusion-weighted MR imaging. *Magn Reson Med* 2003; **50**(5)**:** 1077-1088.

22. Behzadi Y, Restom K, Liau J, Liu TT. A component based noise correction method (CompCor) for BOLD and perfusion based fMRI. *Neuroimage* 2007; **37**(1)**:** 90-101.

23. Forman SD, Cohen JD, Fitzgerald M, Eddy WF, Mintun MA, Noll DC. Improved assessment of significant activation in functional magnetic resonance imaging (fMRI): use of a cluster-size threshold. *Magn Reson Med* 1995; **33**(5)**:** 636-647.

24. King DW, Leskin GA, King LA, Weathers FW. Confirmatory factor analysis of the clinician-administered PTSD Scale: Evidence for the dimensionality of posttraumatic stress disorder. *Psychological Assessment* 1998; **10**(2)**:** 90.
